# The complete chloroplast genome sequence of *Pinus cembra* L. (Pinaceae)

**DOI:** 10.1080/23802359.2019.1693297

**Published:** 2019-11-22

**Authors:** Thomas Schott, Hilke Schroeder, Katrin Schöning-Stierand, Birgit Kersten

**Affiliations:** Institute of Forest Genetics, Thünen Institute, Grosshansdorf, Germany

**Keywords:** Chloroplast genome, genome assembly, Pinaceae

## Abstract

The Swiss pine (*Pinus cembra*) is a montane tree in Central Europe and, therefore, known for its hardiness against severe winter colds. The seeds are harvested and eaten as pine nuts. We assembled and characterized the complete chloroplast genome of *P. cembra* to serve as a valuable resource in future genetic studies. The complete plastome sequence is 116,609 bp in length and contains 113 genes including 79 protein-coding genes, 30 tRNA genes, and 4 rRNA genes. A phylogenetic analysis of 34 *Pinus* plastome sequences shows that *Pinus sibirica* is the nearest relative to *P. cembra* and that there is a distinct clustering together with the other members of the section Quinquefoliae.

*Pinus cembra*, also known as Swiss pine, is a conifer tree in the *Pinus* genus (family Pinaceae) that grows in the Alps and Carpathian Mountains of central Europe at 1200–2300 m altitude. The slowly growing trees of this species are long-lasting and can reach an age between 500 and 1000 years. *Pinus cembra* is a member of the subgenus Strobus (white pine group) – one of the two subgenera in the genus. The complete chloroplast genome sequence of *Pinus cembra* represents the first genome sequence resource for this species and will extend the existing organelle genome resources, currently comprising chloroplast genome sequences of 33 species and the mitochondrial genome sequence of *Pinus taeda* (https://www.ncbi.nlm.nih.gov/genome/organelle/).

The reference specimen (PICEM_1_1) was selected for sequencing from the Arboretum of the Thuenen Institute of Forest Genetics, Grosshansdorf (Arboretum number 122/CIII). Needles were sampled and DNA (Voucher specimen: sample accession PICEM_1_1; stored at the Thuenen Institute of Forests Genetics) was extracted according to Dumolin et al. (1995). Standard genomic library preparation and 150 bp paired-end sequencing were performed using Illumina HiSeq 4000 (Illumina, San Diego, CA, USA) at 1× haploid genome coverage (GATC Biotech AG, A Eurofins Genomics Company, Konstanz, Germany). Reads were adaptor-clipped using MIRA (Chevreux et al. 1999) and assembled using NOVOplasty version 3.6 (Dierckxsens et al. 2017) with the partial *P. cembra rbcL* gene as seed (NCBI Genbank acc. DQ353720.1) and the *Pinus sibirica* chloroplast genome as reference (NCBI NC_028552.2). Reads mapping to any of the possible assemblies (bowtie2; Langmead and Salzberg 2012) were reassembled using SPAdes version 3.12 (−k = 55,89,127) (Bankevich et al. 2012). Resulting contigs were ordered and scaffolded using Mauve version 2.4.0 (Darling et al. 2010) and the *P. sibirica* chloroplast genome as reference. Overlapping contigs were manually joined; remaining gaps were closed using GapFiller version 1.9 (Boetzer and Pirovano 2012). The resulting circular sequence was functionally annotated using the GeSeq server (Tillich et al. 2017). The complete chloroplast genome sequence of *P. cembra* (Genbank MN536531) has a total length of 116,609 bp and consists of a large single-copy region (63,891 bp), a small single-copy region (51,722 bp), and two inverted repeat regions (473 bp, each). The annotated sequence contains 113 genes including 79 protein-coding genes, 30 tRNA genes, and 4 rRNA genes. The CG content of the complete sequence averages 38.7%.

A phylogenetic tree (Figure 1) was created based on multiple sequence alignment (CLC-GWB version 12, Qiagen Aarhus, Denmark) of 34 complete chloroplast DNA sequences of *Pinus* species from both subgenera *Pinus* and *Strobus*. *Larix sibirica* (Pinales, Pinaceae) served as an out-group using artificially rearranged conserved sequence blocks of the chloroplast DNA sequence to match the synteny observed within the genus *Pinus*.

**Figure 1.**
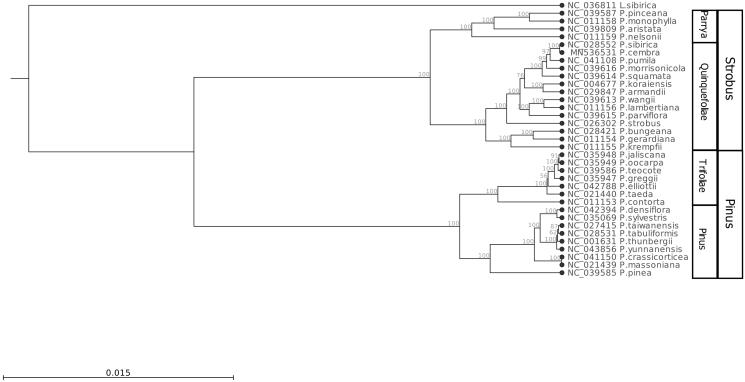
Phylogenetic tree (maximum likelihood) based on whole-plastome sequences of *Pinus species and Larix sibirica* (outgroup). Bootstrap values (%) are shown above branches. The phylogenetic tree was constructed based on a whole-plastome alignment using the ‘Maximum likelihood phylogeny’ tool of CLC-GWB including bootstrap analysis with 100 replicates (other parameters: construction method for the start tree = UPGMA; nucleotide substitution model = Jukes–Cantor). The assignment of the *Pinus* species to the two subgenera Pinus and Strobus and related sections is provided on the right side.

## References

[CIT0001] Bankevich A, Nurk S, Antipov D, Gurevich A, Dvorkin M, Kulikov AS, Lesin V, Nikolenko S, Pham S, Prjibelski A, et al. 2012 SPAdes: a new genome assembly algorithm and its applications to single-cell sequencing. J Comp Biol. 19(5):455–477. 10.1089/cmb.2012.0021 PMC3342519 22506599

[CIT0002] Boetzer M, Pirovano W 2012 Towards almost closed genomes with GapFiller. Genome Biol. 13(6):R56. 22731987 10.1186/gb-2012-13-6-r56 PMC3446322

[CIT0003] Chevreux B, Wetter T, Suhai S, 1999 Genome sequence assembly using trace signals and additional sequence information. Comput Sci Biol: Proceedings of the German Conference on Bioinformatics (GCB). 99:45–56.

[CIT0004] Darling AE, Mau B, Perna NT 2010 Progressive mauve: multiple genome alignment with gene gain, loss and rearrangement. PLOS One. 5(6):e11147. 20593022 10.1371/journal.pone.0011147 PMC2892488

[CIT0005] Dierckxsens N, Mardulyn P, Smits G 2017 NOVOPlasty: de novo assembly of organelle genomes from whole genome data. Nucleic Acids Res. 45(4):e18. 28204566 10.1093/nar/gkw955 PMC5389512

[CIT0006] Dumolin S, Demesure B, Petit RJ 1995 Inheritance of chloroplast and mitochondrial genomes in pedunculate oak investigated with an efficient PCR method. Theor Appl Genet. 91(8):1253–1256. 24170054 10.1007/BF00220937

[CIT0007] Langmead B, Salzberg SL 2012 Fast gapped-read alignment with Bowtie 2. Nat Methods. 9(4):357–359. 22388286 10.1038/nmeth.1923 PMC3322381

[CIT0008] Tillich M, Lehwark P, Pellizzer T, Ulbricht-Jones ES, Fischer A, Bock R, Greiner S 2017 GeSeq – versatile and accurate annotation of organelle genomes. Nucleic Acids Res. 45(W1):W6–W11. 28486635 10.1093/nar/gkx391 PMC5570176

